# Supplementary material for: Mitochondrially targeted proximity biotinylation and proteomic analysis in Plasmodium falciparum
Source: PLoS One. 2022 Aug 19;17(8):e0273357. doi: 10.1371/journal.pone.0273357 (PMC9390924; doi:10.1371/journal.pone.0273357)

Figure 1B

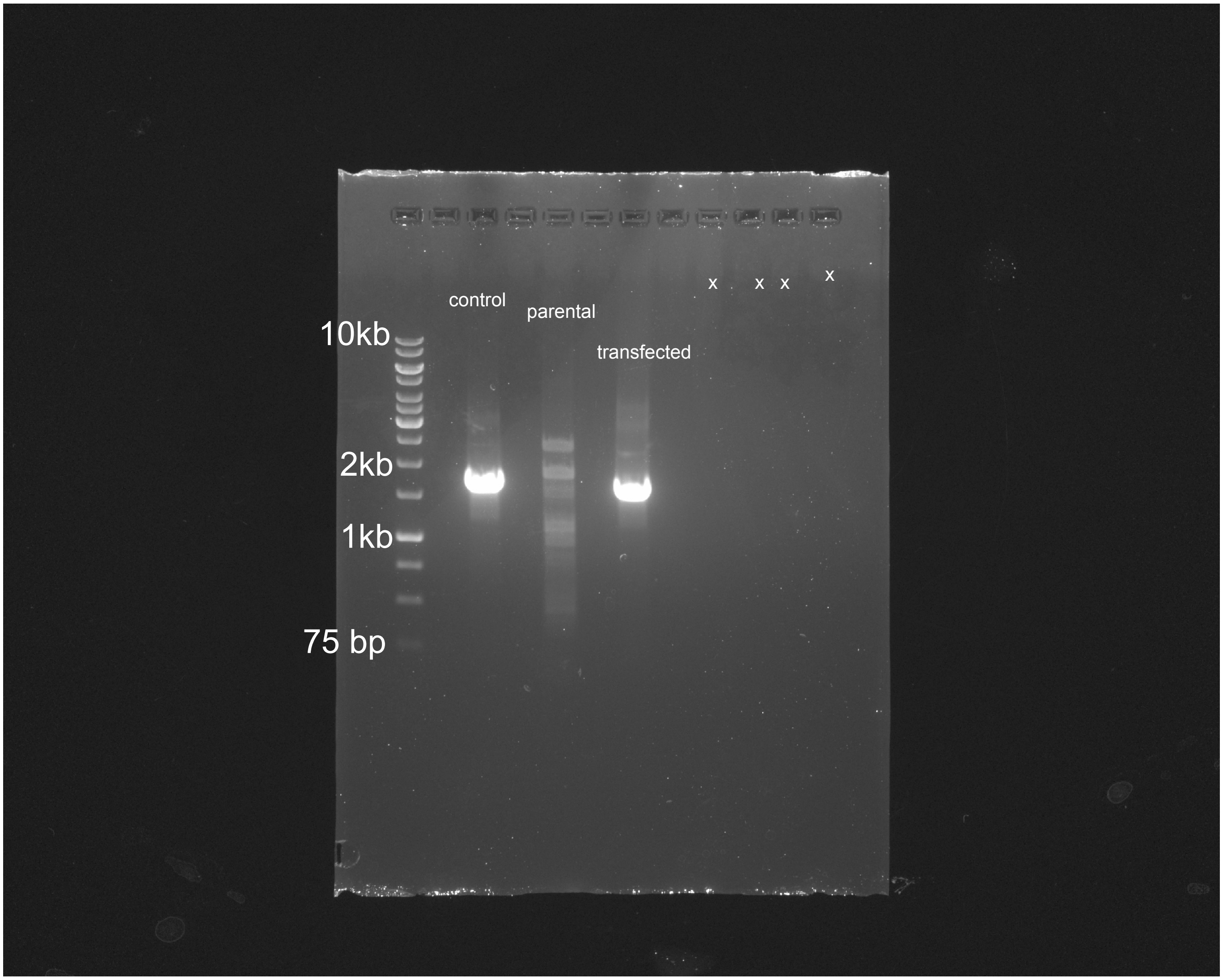

Figure 1D Anti-HA

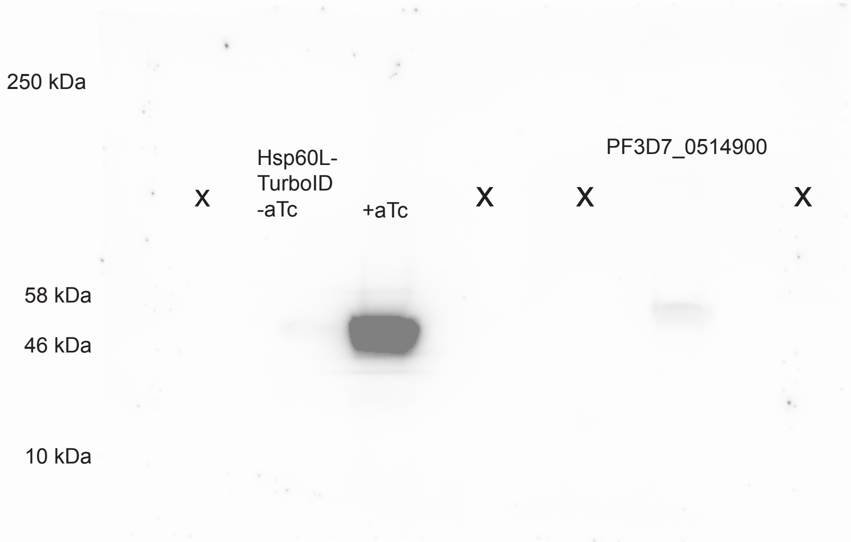

Figure 1D Loading Control for HSP60L TurboID-- Anti-Exp2

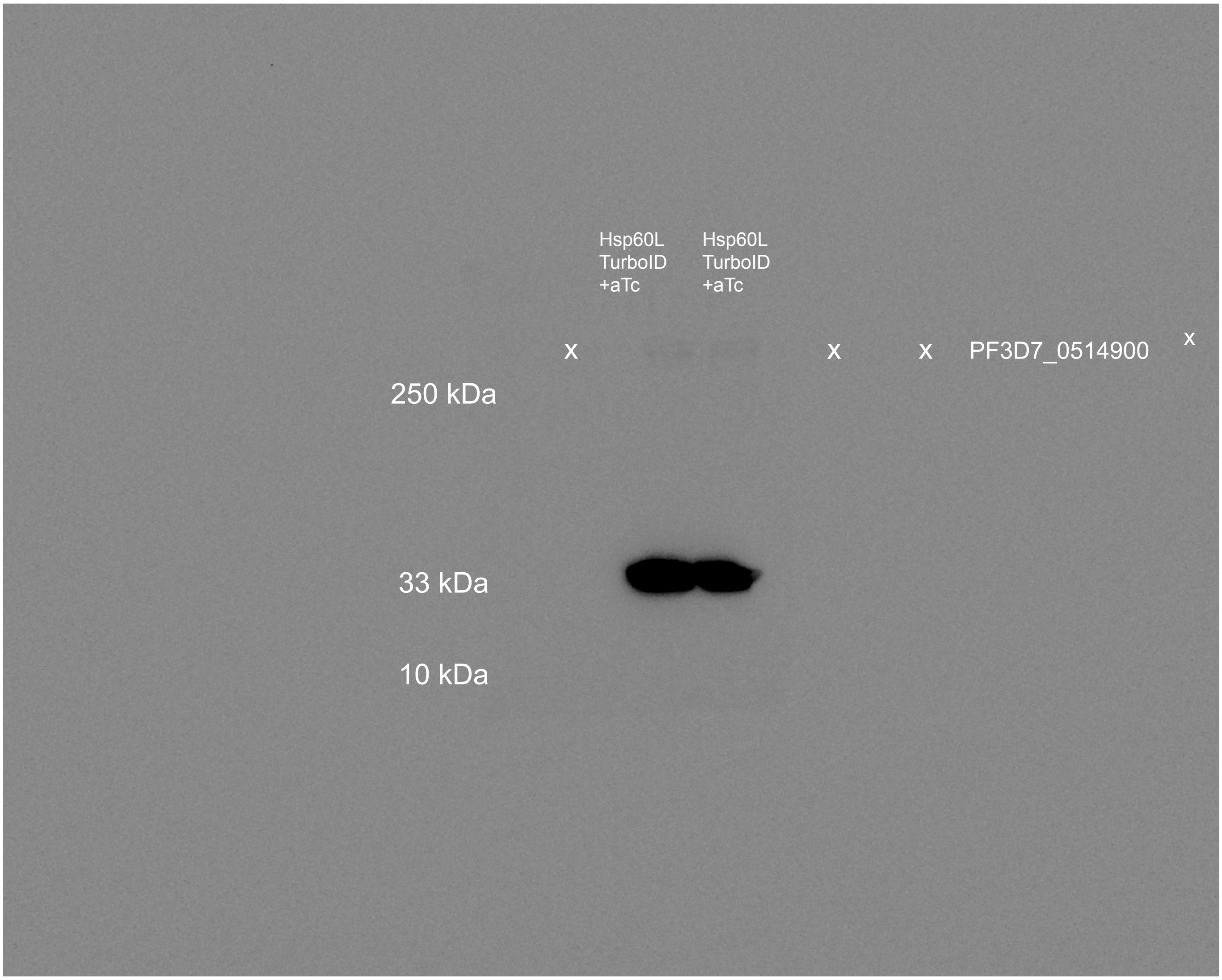

Figure 2A

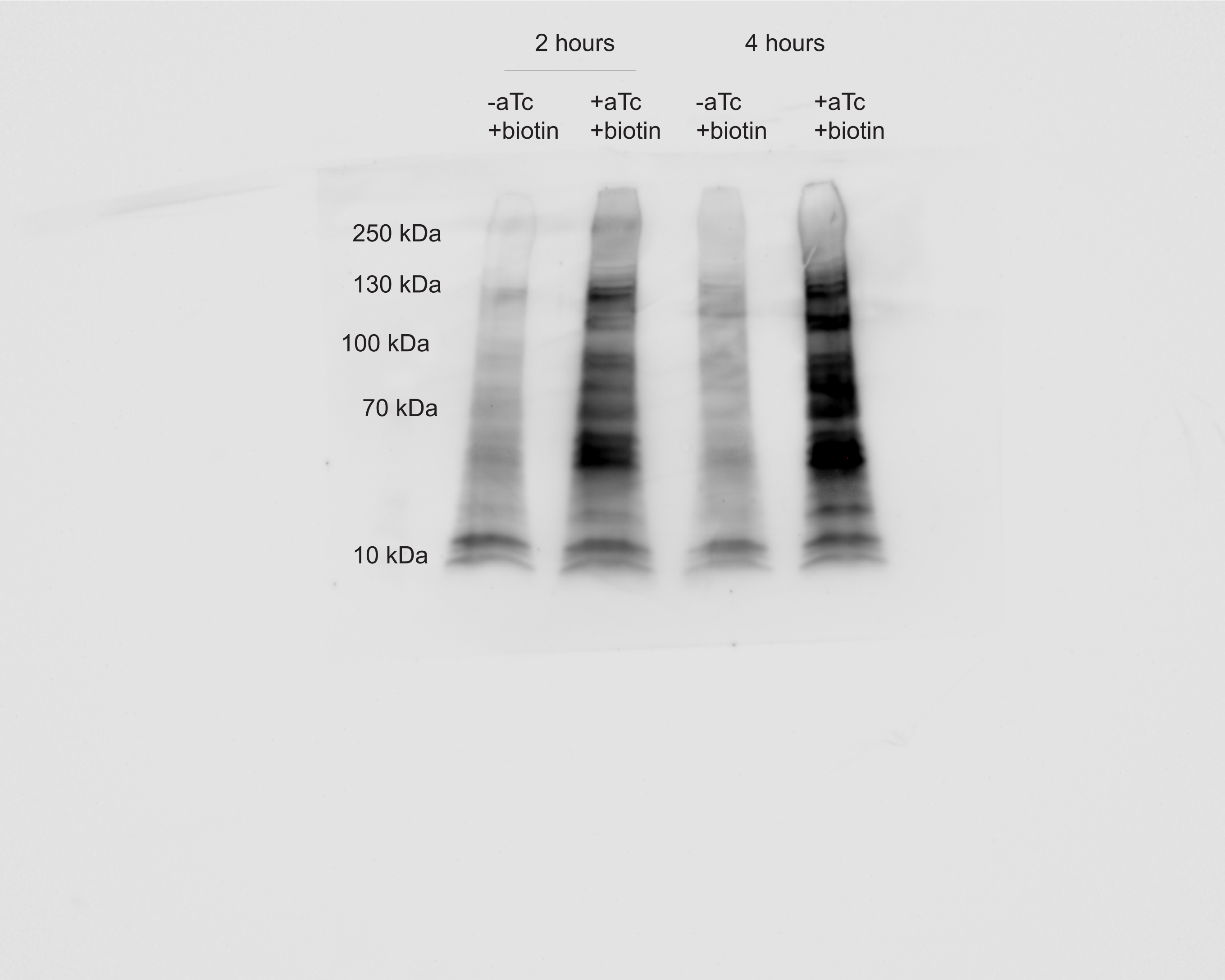
2 hours
4 hours
-aTc
+biotin
+aTc
+biotin
-aTc
+biotin
+aTc
+biotin
250 kDa
130 kDa
100 kDa
70 kDa
10 kDa

Figure 3B

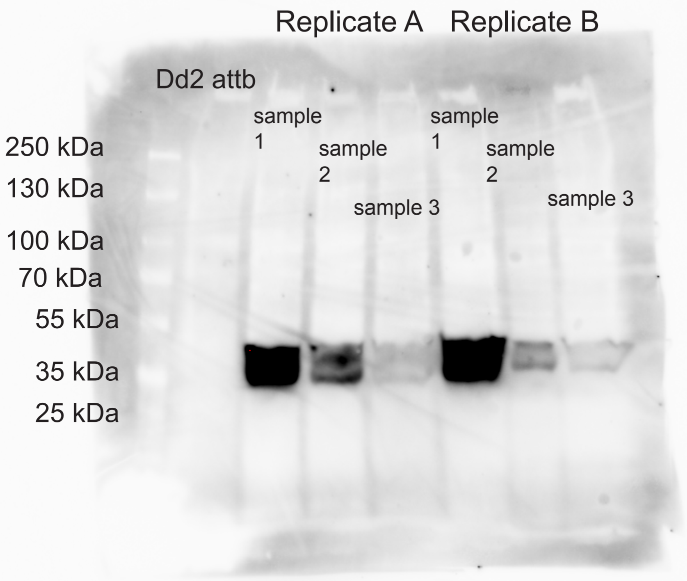

Replicate A
Replicate B
Dd2 attb
sample 1
sample 2
sample 3
sample 1
sample 2
sample 3
250 kDa
130 kDa
100 kDa
70 kDa
55 kDa
35 kDa
25 kDa

Figure 4A

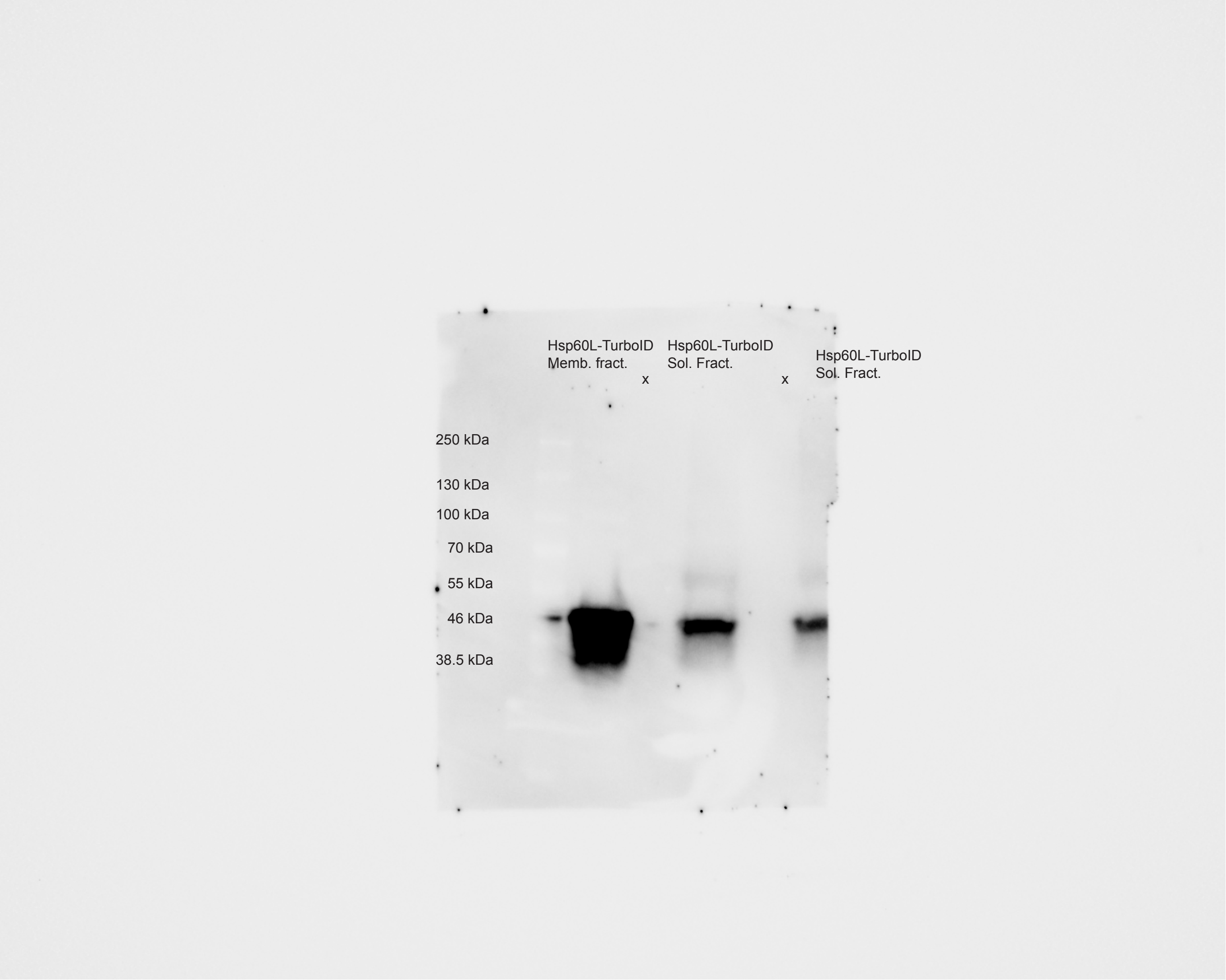
Hsp60L-TurboID
Memb. fract.
x
Hsp60L-TurboID
Sol. Fract.
x
Hsp60L-TurboID
Sol. Fract.
250 kDa
130 kDa
100 kDa
70 kDa
55 kDa
46 kDa
38.5 kDa

Figure 5 Anti-HA

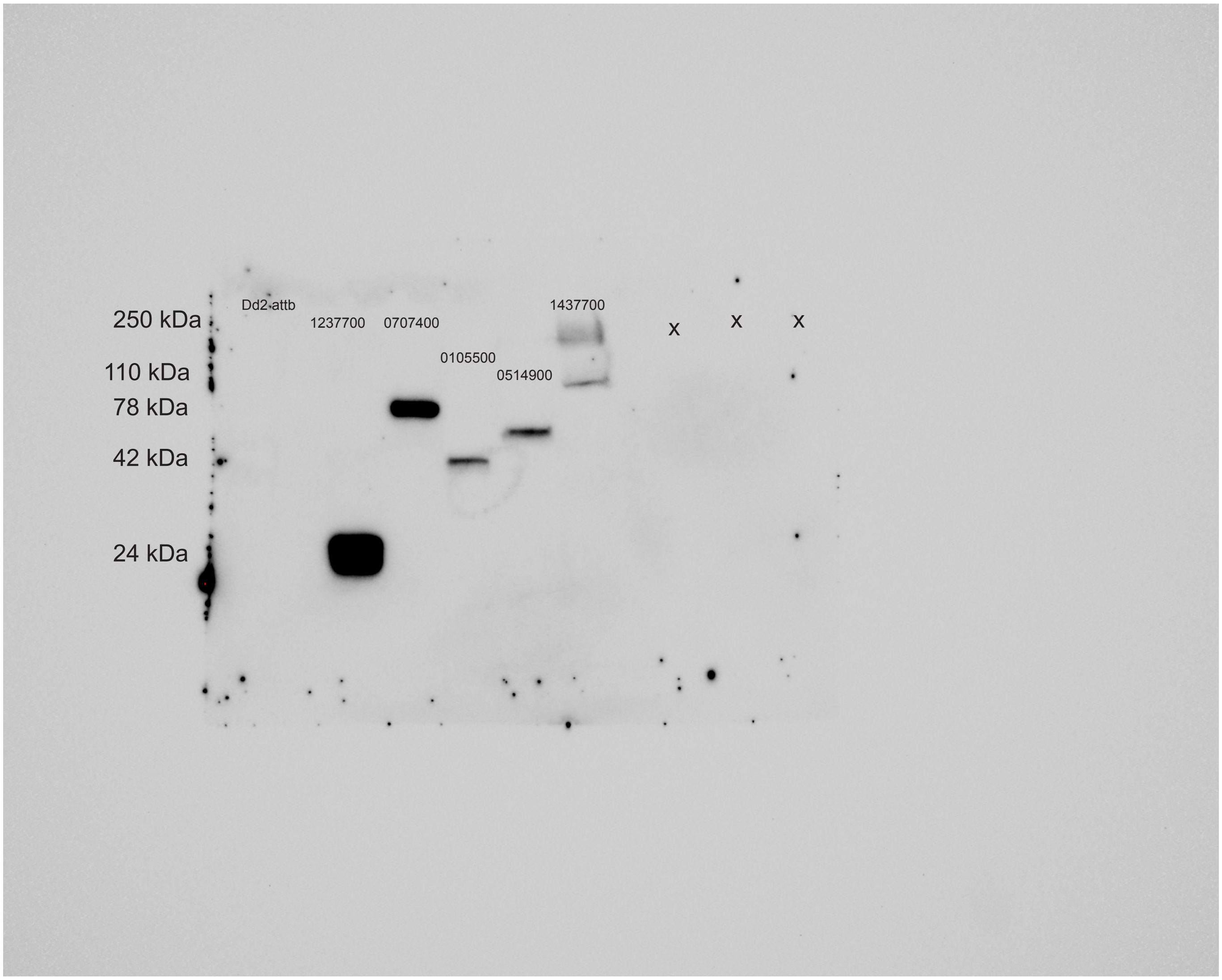

Figure 5 Anti-Aldolase

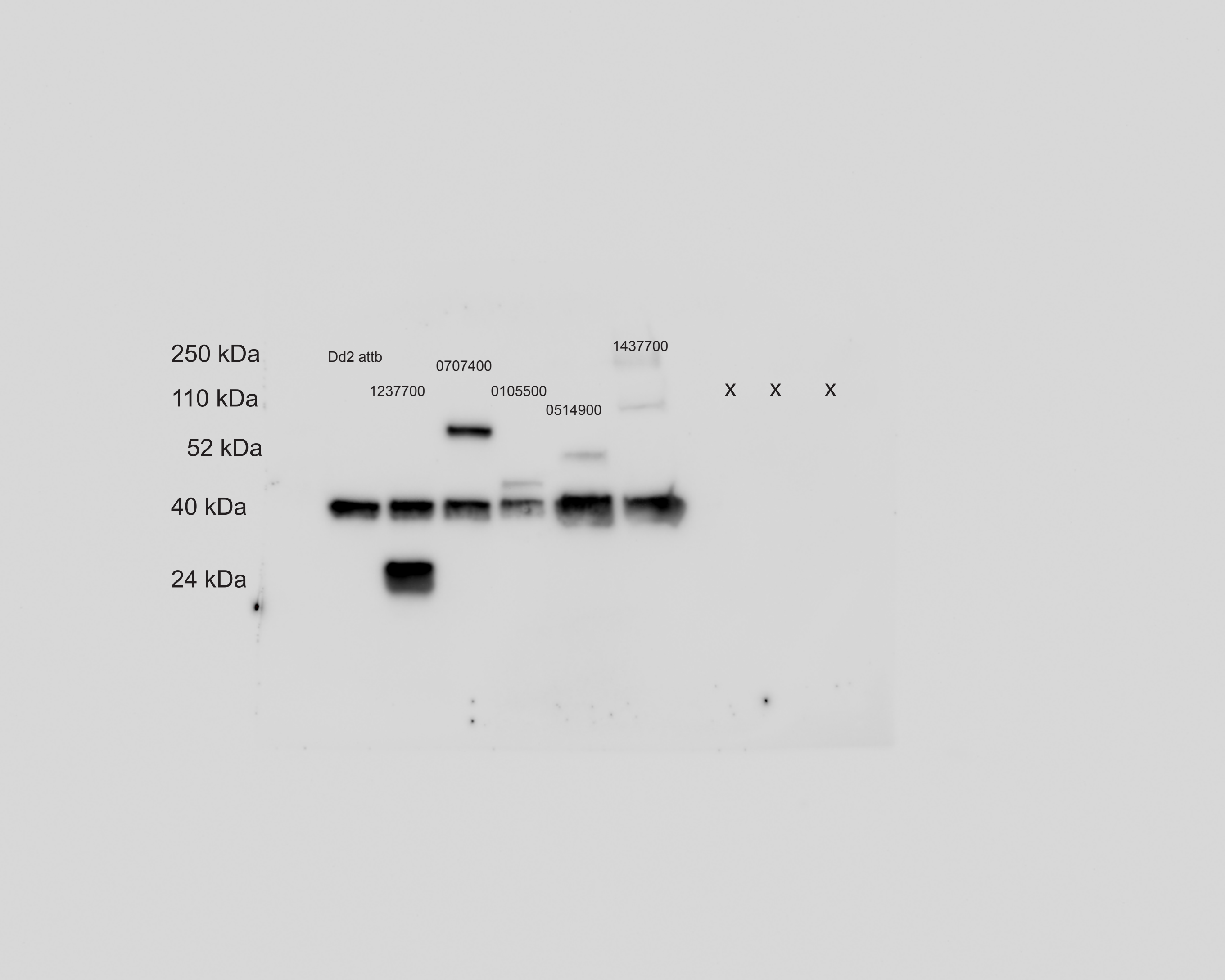

Supplement: S1 Raw images — (PDF) [file pone.0273357.s006.pdf]
